# The first meta-analysis of the G96S single nucleotide polymorphism (SNP) of the prion protein gene (*PRNP*) with chronic wasting disease in white-tailed deer

**DOI:** 10.3389/fvets.2024.1437189

**Published:** 2024-11-29

**Authors:** Sae-Young Won, Yong-Chan Kim

**Affiliations:** Department of Biological Sciences, Andong National University, Andong, Republic of Korea

**Keywords:** prion, PRNP, PrP^Sc^, scrapie, BSE, CWD, CJD, white-tailed deer

## Abstract

**Background:**

Prion diseases are irreversible infectious neurodegenerative diseases caused by a contagious form of prion protein (PrP^Sc^). Since chronic wasting disease (CWD)-infected white-tailed deer are strong carriers of the prion seed through corpses via scavenger animals, preemptive control based on genetic information for a culling system is necessary. However, the risk of CWD-related genetic variants has not been fully evaluated. In the present study, we carried out a quantitative estimation of the risk of a G96S single nucleotide polymorphism (SNP) of the *PRNP* gene to CWD infection in white-tailed deer.

**Methods:**

We carried out a literature search for genetic data of the G96S (c.286G>A) SNP of the *PRNP* gene from CWD-infected white-tailed deer and matched controls. We performed a meta-analysis using incorporated eligible studies to evaluate the association of the G96S SNP of the *PRNP* gene with susceptibility to CWD in white-tailed deer.

**Results:**

We identified a strong association between the G96S (c.286G>A) SNP of the *PRNP* gene and susceptibility to CWD infection in white-tailed deer using meta-analysis. We observed the most significant association in the recessive model (odds ratio = 3.0050, 95% confidence interval: 2.0593; 4.3851, *p* < 0.0001), followed by the additive model (odds ratio = 2.7222, 95% confidence interval: 1.9028; 3.8945, *p* < 0.0001) and the heterozygote (AA vs. AG) comparison (odds ratio = 2.7405, 95% confidence interval: 1.9215; 3.9085, *p* < 0.0001).

**Conclusion:**

To the best of our knowledge, this was the first meta-analysis of the association between the G96S (c.286G>A) SNP of the *PRNP* gene and susceptibility to CWD infection.

## 1 Introduction

Prion diseases are malignant contagious neurodegenerative diseases caused by an infectious form of prion protein (PrP^Sc^) converted from a benign form of prion protein (PrP^C^) (1–3). Several types of prion diseases have been reported worldwide including scrapie in sheep and goats; bovine spongiform encephalopathy (BSE) in cattle; Creutzfeldt-Jakob disease (CJD), fatal familial insomnia (FFI), Gerstmann–Straussler–Scheinker syndrome (GSS), and kuru in humans; and chronic wasting disease (CWD) in the Cervidae family (2, 4, 5). PrP^Sc^ acts as a conversion seed and changes PrP^C^ to a ß-sheet-rich aggregate. The aggregate accumulates in brain lesions and is accompanied by several neurotoxic symptoms including neuron loss, vacuolation, and astrogliosis (1, 6, 7). During the conversion process, genetic variants of the amino acid sequences of prion protein (PrP), which are encoded by the prion protein gene (*PRNP*), play a pivotal role in susceptibility to prion diseases (8, 9). In humans, the M129V single nucleotide polymorphism (SNP) provides resistance to CJD (10). In addition, the G127V natural genetic variant blocks kuru (11). In sheep, scrapie susceptibility is modulated by haplotypes located on codons 136, 154, and 171 (12). In goats, codons at 142, 146, 154, 211, and 222 of the *PRNP* gene contribute to prion disease resistance (12–14). Like other prion diseases, CWD hosts have unique susceptibility-related genetic variants (5, 15). In elk, M132L SNP is associated with CWD propagation (16). In sika deer, a study indicated that S19N is related to CWD vulnerability (17). In white-tailed deer, several studies have suggested that G96S is the most potent candidate for prion-related genetic factors (18–27). However, quantitative studies have not been performed.

Among the several types of prion disease, CWD is the most infectious because it can be vertically transmitted, as through CWD prion-contaminated soil (28). Therefore, preemptive control with a culling system using genetic information is a crucial step to prevent the extensive spread of CWD. Since some CWD-infected white-tailed deer are free-ranging, there is a greater possibility of prion contamination through corpses via scavenger animals and of the emergence of novel prion hosts and strains (29). In addition, older free-ranging deer of indeterminate slaughter age carrying germline and somatic mutations in the *PRNP* gene are more likely to develop prion disease. Since CWD seed inducible genetic variants have not been fully elucidated, a quantitative evaluation of the connection between G96S of the *PRNP* gene and CWD susceptibility in white-tailed deer is necessary.

In the present study, we searched the literature for study data on the genotype and allele distributions of the G96S SNP of the *PRNP* gene from CWD-infected white-tailed deer and matched controls to evaluate the association between the G96S SNP of the *PRNP* gene and susceptibility to CWD in white-tailed deer. Then, we carried out a meta-analysis using incorporated eligible studies.

## 2 Materials and methods

### 2.1 Search strategy

We carried out the meta-analysis according to the Preferred Reporting Items for Systematic Reviews and Meta-Analysis (PRISMA) guidelines. A literature search was conducted in the PubMed, Google Scholar, and Web of Science databases to find studies associated with the G96S (c.286G>A) SNP of the *PRNP* gene from CWD-infected white-tailed deer. The following terms were employed for the search: “*PRNP*,” “white-tailed deer,” and “CWD” or “Chronic wasting disease” combined with “polymorphism” or “SNP” (last search update: March 12, 2024).

Eligible studies adhered to the subsequent inclusion criteria: (1) relating to and containing the association between the G96S (c.286G>A) SNP and CWD-infected white-tailed deer; (2) a case-control study; (3) full text available; and (4) written in English. The exclusion criteria were as follows: (1) case reports and (2) insufficient genetic information.

### 2.2 Meta-analysis

The meta-analysis was performed using the meta package of the R program [https://www.r-project.org/ (accessed on March 12, 2024)] and involved calculation of the pooled odds ratios with 95% confidence intervals between the G96S (c.286G>A) SNP of the *PRNP* gene and susceptibility to CWD. We utilized a total of 7 genetic models: additive (A vs. G), recessive (AA vs. AG + GG), dominant (AA + AG vs. GG), and over-dominant (AG vs. AA + GG) models and homozygote (AA vs. GG) and heterozygote (AA vs. AG and AG vs. GG) comparisons. Heterogeneity was calculated using the *p*-value and *I*^2^ value. The fixed and random effect models were selected according to the value of the *I*^2^ test. Publication bias was estimated using Egger’s weighted regression methods.

## 3 Results

### 3.1 Study selection

**Figure 1:**
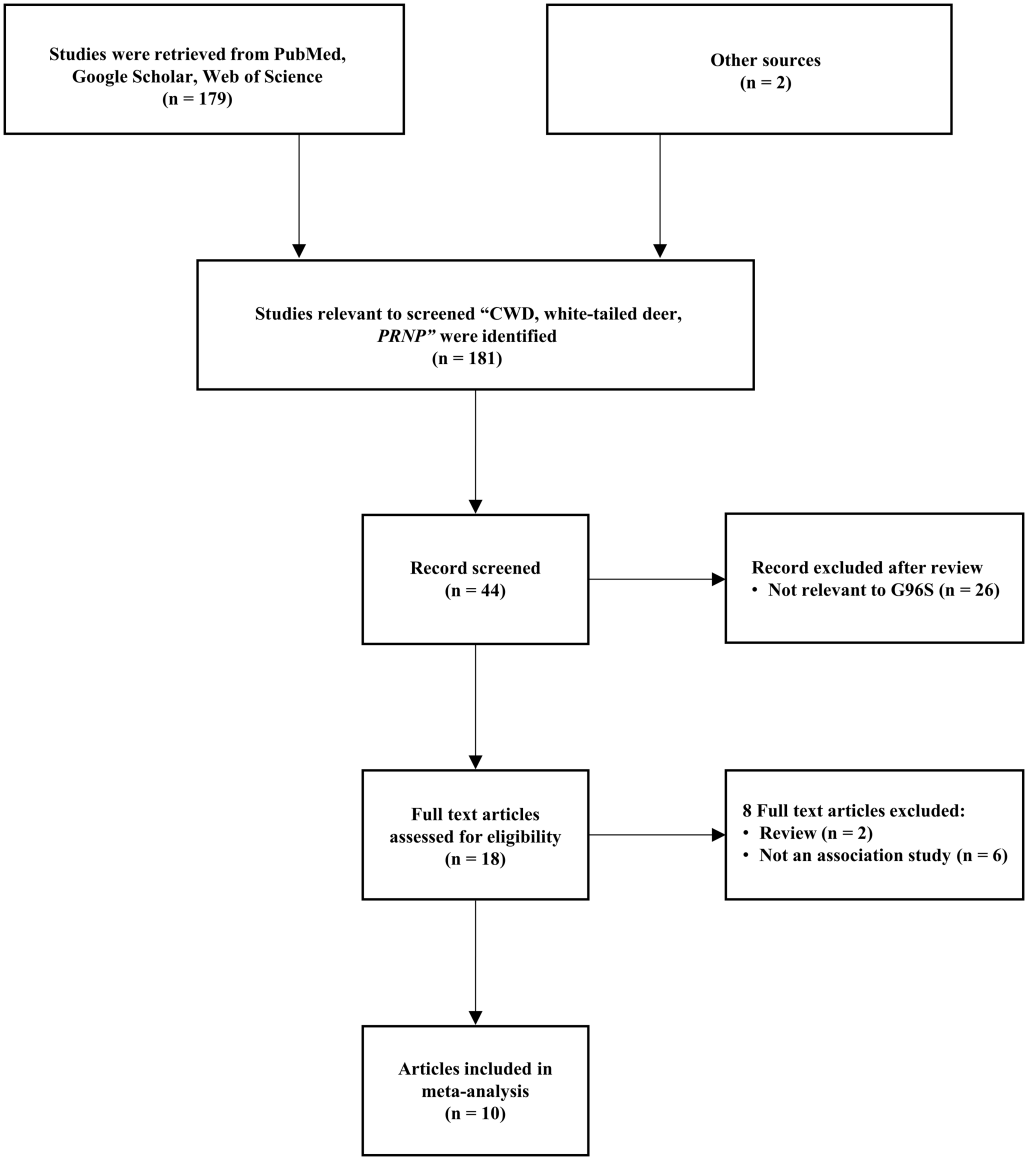
Flow chart of the study selection process.

A flow chart of the study selection process is shown in Figure 1. After excluding ineligible articles based on the inclusion and exclusion criteria, 10 relevant studies were extracted from the databases (PubMed, Google Scholar, Web of Science). The meta-analysis comprised 1,003 cases and 1,335 controls (Table 1). All eligible studies utilized a case–control comparison using two genotyping methods. Among the 10 studies, 2 studies were genotyped using polymerase chain reaction (PCR)-restriction fragment length polymorphism (RFLP) methods. The remaining 8 studies were genotyped using PCR-amplicon sequencing methods (Table 1).

**Table 1:** Characteristics of studies included in the meta-analysis.

Study	Year	State	Nation	Genotyping methods	Total sample size	Number of controls	Number of cases
Chad et al.	2003	Wisconsin	USA	RFLP	120	94	26
Katherine et al.	2004	Nebraska	USA	RFLP	131	64	67
Chad et al.	2006	Wisconsin	USA	Sequencing	445	153	292
Delwyn et al.	2008	Wisconsin	USA	Sequencing	73	14	59
Amy et al.	2008	Illinois	USA	Sequencing	196	120	76
Gregory et al.	2009	Alberta, Saskatchewan	Canada	Sequencing	227	197	30
Julie et al.	2009	Wisconsin	USA	Sequencing	137	72	65
Adam et al.	2015	Illinois, Wisconsin	USA	Sequencing	240	135	105
Robert et al.	2020	Nebraska	USA	Sequencing	201	103	98
Caitlin et al.	2021	Michigan	USA	Sequencing	568	383	185
Total	—	—	—	—	2,338	1,335	1,003

### 3.2 Evaluation of the association between the G96S (c.286G>A) SNP of the *PRNP* gene and susceptibility to CWD by meta-analysis in white-tailed deer

The pooled, weighted genotyping results were combined according to sample size to evaluate the association between the G96S SNP of the *PRNP* gene and susceptibility to CWD using the meta package of the R program. The pooled odds ratios with 95% confidence intervals were evaluated based on 7 genetic models: additive (A vs. G), recessive (AA vs. AG + GG), dominant (AA + AG vs. GG), and over-dominant (AG vs. AA + GG) models and homozygote (AA vs. GG) and heterozygote (AA vs. AG and AG vs. GG) comparisons. Heterogeneity was estimated using the *p*-value and *I*^2^ value (Table 2). The dominant model (AA + AG vs. GG) and homozygote (AA vs. GG) and heterozygote (AG vs. GG) comparisons used fixed models according to the *I*^2^ value (>0.5). The remaining 4 genetic models used random models according to the *I*^2^ value (<0.5).

**Table 2:** Meta-analysis of the association between G96S (c.286G>A) of the prion protein gene (*PRNP*) gene and susceptibility to chronic wasting disease (CWD).

Genetic model	Association test			Heterogeneity			Publication bias
	Odds ratio	95% confidence interval	*p*-value	Model	*p*-value	*I* ^2^	Egger’s test *p*-value
Additive model (A vs. G)	2.7222	[1.9028; 3.8945]	2.963 ×10^−7^	Random	<0.01	0.70	0.2078
Recessive model (AA vs. AG + GG)	3.0050	[2.0593; 4.3851]	8.09 ×10^−8^	Random	<0.01	0.64	0.4141
Dominant model (AA + AG vs. GG)	2.4081	[1.3951; 4.1568]	0.0112199714	Fixed	0.18	0.29	0.0065
Over-dominant model (AG vs. AA + GG)	0.4067	[0.2817; 0.5871]	1.0949 ×10^−5^	Random	0.01	0.59	0.7684
AA vs. GG	2.9883	[1.7221; 5.1856]	0.0006936882	Fixed	0.11	0.38	0.0043
AA vs. AG	2.7405	[1.9215; 3.9085]	1.827 ×10^−7^	Random	0.02	0.55	0.6524
AG vs. GG	1.3313	[0.7495; 2.3650]	1	Fixed	0.29	0.16	0.0535

Except for the heterozygote (AG vs. GG) comparison, the data from the 9 remaining genetic models showed a strong association between the risk of CWD and the G96S (c.286G>A) SNP of the *PRNP* gene in the additive model (odds ratio = 2.7222, 95% confidence interval: 1.9028; 3.8945, *p* < 0.0001), recessive model (odds ratio = 3.0050, 95% confidence interval: 2.0593; 4.3851, p < 0.0001), dominant model (odds ratio = 2.4081, 95% confidence interval: 1.3951; 4.1568, *p* < 0.05), over-dominant model (odds ratio = 0.4067, 95% confidence interval: 0.2817; 0.5871, *p* < 0.001), homozygote comparison (odds ratio = 2.9883, 95% confidence interval: 1.7221; 5.1856, *p* < 0.001), and heterozygote (AA vs. AG) comparison (odds ratio = 2.7405, 95% confidence interval: 1.9215; 3.9085, *p* < 0.0001).

**Figure 2:**
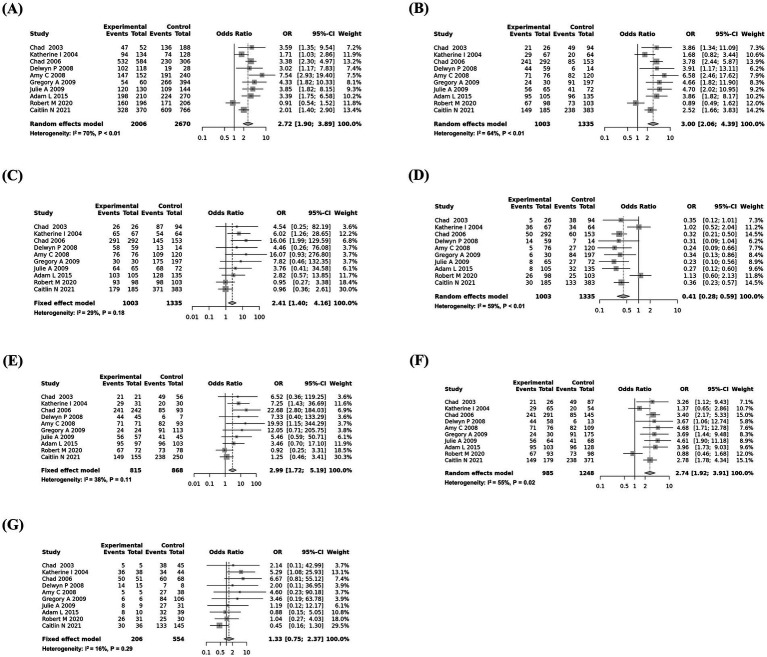
Forest plots of the association between the G96S (c.286G>A) single-nucleotide polymorphism (SNP) of the prion protein gene (*PRNP*) gene and susceptibility to chronic wasting disease (CWD). **(A)** Additive model (A vs. G). **(B)** Recessive model (AA vs. AG + GG). **(C)** Dominant model (AA + AG vs. GG). **(D)** Over-dominant model (AG vs. AA + GG). **(E)** Homozygote comparison (AA vs. GG). **(F)** Heterozygote comparison (AA vs. AG). **(G)** Heterozygote comparison (AG vs. GG).

To estimate potential publication bias, Egger’s tests were carried out, and publication bias was observed in the dominant model and homozygote (AA vs. GG) and heterozygote comparisons (AG vs. GG). The details of the values are described in Table 2. Except for publication bias that violated genetic models, the most significant association was observed in the recessive model, followed by the additive model and heterozygote (AA vs. AG) comparison. Forest plots are shown in Figure 2.

## 4 Discussion

White-tailed deer are raised on farms or live in the wild, and CWD-infected white-tailed deer have been reported in the USA and Canada in North America (23, 30). In previous surveillance for prion diseases in Wisconsin, field cases were reported in several scavenger animals including mink, raccoon, and opossums (29). The results of that study indicated that free-ranging CWD-infected white-tailed deer are strong prion carriers and possible CWD pandemic inducers in the ecosystem. In addition, recent studies have indicated that a sporadic type of animal prion disease, atypical BSE, was identified in old animals (8 years old or older) (31). Since sporadic human prion diseases account for over 85% of the total number (32), and the slaughter age is not determined for free-ranging white-tailed deer, the possibility of sporadic occurrence may be much higher in white-tailed deer than in other farmed animals. Therefore, it is important to investigate CWD-related genetic factors for use in a culling system for preemptive prevention of CWD in white-tailed deer.

Previous genome-wide association analysis (GWAA) suggested that G96S is the large-effect risk locus; however, that study did not provide the quantitative value of the CWD risk (33). In the present study, we quantitatively evaluated the risk of the G96S SNP of the *PRNP* gene for CWD in white-tailed deer and observed a strong association in the recessive (AA vs. AG + GG) and additive (A vs. G) models and the heterozygote (AA vs. AG) comparison (Table 2). In summary, the 96G allele confers a risk of CWD that is more than 2.5 times higher than that of the 96S allele in these genetic models (Table 2).

Codon 96 is located on the unstructured coil region between the octapeptide repeat region and ß-sheet of cervid PrP (15). Several pathogenic mutations including P102L, P105L, and A117V of human PrP were identified in a similar region (34). Thus, this region seems to play a pivotal role in the conversion of PrP^Sc^. However, although M129V heterozygotes provided potent resistance to CJD, human PrP with the 129M allele showed a similar structure and characteristics to human PrP with the 129V allele. The prion-resistant effect of the 129V allele is related to the homotypic amino acid sequence between the template protein and prion seed (PrP^Sc^) (35). In addition, the results of a recent real-time quaking-induced conversion (RT-QuIC) study indicated that conversion efficiency (PrP^C^ to PrP^Sc^) is significantly related to the correspondence of the amino acid sequence at codon 132 of the elk PrP between the seed (PrP^Sc^) and template protein (36). Thus, further investigation of the effects of codon 96 on seed-template interaction is required to elucidate the pathomechanism of codon 96. In white-tailed deer, several reports have indicated that codon 95 is also associated with susceptibility to CWD (15, 18). In addition, a recent study suggested that codon 116 is related to the stability of CWD prion, sensitivity to proteinase K, and lower prion seeding activity (37). Since the present study did not reflect other codons or the haplotypes of the *PRNP* gene, further investigation of genetic polymorphisms at other codons and the haplotypes of the *PRNP* gene are highly desirable in the future. Given the complexity of the pathological mechanisms of CWD, fully understanding them is challenging; therefore, we focused on a single factor (G96S SNP) in this study. However, a comprehensive analysis involving other codons will be necessary in the future to fully capture the phenomenon in real-world settings.

In conclusion, this study is the first meta-analysis to quantitatively assess the G96S SNP’s association with CWD susceptibility in white-tailed deer.

## Data Availability

The original contributions presented in the study are included in the article/supplementary material, further inquiries can be directed to the corresponding author.
